# Depletion of gut microbiota improves the therapeutic efficacy of cancer nanomedicine

**DOI:** 10.7150/thno.73873

**Published:** 2022-10-17

**Authors:** Ray Putra Prajnamitra, Yuan-Yuan Cheng, Chaw Yee Beh, Chien-Yi Lu, Jen-Hao Lin, Shu-Chian Ruan, Sheng-Lun Chen, Hung-Chih Chen, Ruey-Bing Yang, Patrick Ching-Ho Hsieh

**Affiliations:** 1 Institute of Biomedical Sciences, Academia Sinica, Taipei 115, Taiwan.; 2 Institute of Medical Genomics and Proteomics and Institute of Clinical Medicine, National Taiwan University College of Medicine, Taipei 100, Taiwan.

**Keywords:** Gut microbiota, triple-negative breast cancer, nanomedicine, LipoDox, vascular permeability

## Abstract

**Rationale:** Gut microbiota plays a crucial role in cancer development and treatment. Studies show that although the gut microbiota is able to promote tumor growth, its presence also improves the efficacy of cancer treatment such as immunotherapy. To date, understanding of the potential impact of the gut microbiota on other treatment modalities such as cancer nanomedicine is still limited. In this study, we aimed to establish the relationship between gut microbiota and cancer nanomedicine, which can potentially open a new path in cancer treatment that combines gut microbiota modulation along with nanotherapeutics.

**Methods:** Mice bearing 4T1 triple-negative breast cancer cells were subjected to gut microbiota modulation by antibiotics (ABX) treatment in the drinking water. Mice given normal water was used for control. The effects of ABX treatment towards gut bacteria was studied by RT-qPCR and 16S next generation sequencing of fecal samples. The mice were then subjected to liposomal doxorubicin (LipoDox) treatment and the amount of nanotherapeutics that accumulated in the tumors was quantified. For therapeutic efficacy, the mice were subjected to ABX treatment and given three injections of LipoDox or saline, while the tumor growth was monitored throughout.

**Results:** Analysis of fecal bacterial content showed that ABX treatment resulted in depletion of gut microbiota. Quantification of LipoDox content revealed significantly increased accumulation in ABX tumor compared to control. Compared to LipoDox treatment alone, we found that combined gut microbiota depletion and LipoDox treatment resulted in augmented long-term anti-tumor efficacy and significantly improved median survival compared to LipoDox only (control vs ABX = 58.5 vs 74 days, *p* = 0.0002, n = 10 for both groups), with two mice surviving until the end of the experimental end point without experiencing relapse. We also identified the increase in vascular permeability of ABX-treated tumors correlated to for improved therapeutic efficacy and outcome.

**Conclusion:** We showed that gut microbiota depletion led to enhanced tumor vascular permeability, which allowed a larger amount of LipoDox nanoparticles to accumulate in the tumor, leading to better long-term effects. Our results suggest that gut microbiota modulation may be exploited in combination with available nanomedicine-based therapeutics to improve cancer diagnosis, therapeutic efficacy and outcome.

## Introduction

Gut microbiota has gained attention as a major player contributing to various physiological conditions including cancer. Depletion of the gut microbiota by antibiotics (ABX) has been reported to hinder the growth of primary and metastatic melanoma, and pancreatic and colon cancers by promoting the infiltration of effector T cells into the tumor 1. In cancer therapy, various studies over the past decade have unanimously concluded that the gut microbiota plays an important role in improving the efficacy of immune checkpoint inhibitor-based cancer immunotherapy by priming the T cells and augmenting their response and recruitment 2-7. Other therapeutic strategies, such as cancer nanomedicine, have also contributed considerably to the field of cancer treatment, however, the impact of the gut microbiota on their efficacy and outcome has not yet been established.

Cancer nanomedicine is a treatment strategy that relies on nanovectors for therapeutic delivery. When these nanovectors are systemically administered, the majority fail to reach the tumor and may be cleared through the kidneys, liver or spleen 8-10. Those that do survive the journey to the tumor site still have to extravasate from the blood vessel into the tumor tissue. The ease of extravasation to the tumor is attributed to the enhanced permeability and retention effect. Aberrant angiogenesis in the tumor together with an elevated expression of vascular endothelial growth factor (VEGF) and other factors result in enhanced vascular permeability, while the absence of lymphatic drainage makes the path into the tumor one-way, resulting in nanoparticle retention and accumulation 11-14.

To date, studies have documented the relationship between the gut microbiota and factors that affect drug delivery *in vivo*. For example, the gut microbiota was shown to support the development of the innate immune cells by promoting myelopoiesis and increasing resistance to foreign body infection 15. Two studies independently showed the connection between the gut microbiota and intestinal angiogenesis 16,17. Microbiota recolonization of germ free (GF) mice by a fecal transplant from pathogen-free mice resulted in a decline in blood-brain barrier permeability and up-regulation of tight junction protein expression 18. All of these studies hinted at the potential influence of the gut microbiota on nanoparticle fate *in vivo* and, consequently, on cancer nanomedicine.

The current study aimed to establish the gut microbiota-nanoparticle relationship conclusively. Elucidating this relationship furthers our understanding of the factors affecting cancer nanomedicine and suggests the possible future combination of gut microbiota modulation with cancer nanomedicine to increase therapeutic efficacy.

## Results and Discussion

### Depletion of gut microbiota results in a change of nanoparticle biodistribution profile

**Figure 1:**
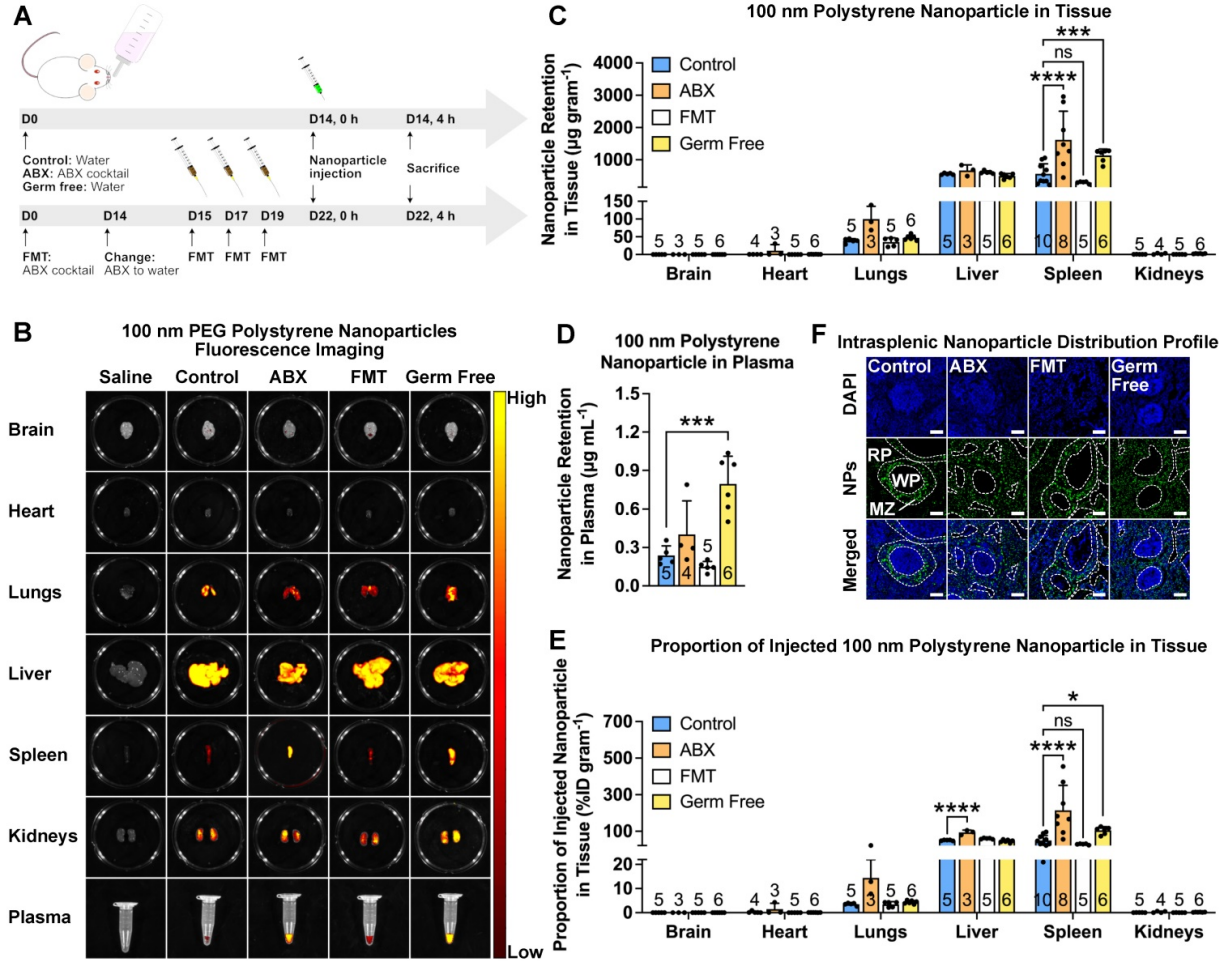
** Gut microbiota modulates nanoparticle biodistribution *in vivo*.** (**A**) Mice were subjected to gut microbiota content modulation prior to systemic nanoparticle administration. Control and germ free mice were given normal drinking water. ABX mice were given ABX cocktail in the drinking water throughout the experiment. FMT mice were given ABX cocktail for two weeks followed by normal water and fecal transplant. PEGylated polystyrene nanoparticles (40 µg gram^-1^ body weight) were administered by intravenous injection *via* the tail vein. (**B**) Representative IVIS images of the major organs and plasma of different groups of mice. (**C-E**) HPLC quantification of 100 nm PEGylated polystyrene nanoparticle content in the major organs and plasma, expressed as nanoparticle content in (**C**) each organ and (**D**) plasma as well as (**E**) the proportion of injected nanoparticles. (**F**) Representative confocal laser scanning microscopy images of intrasplenic nanoparticle distribution. WP: white pulp, RP: red pulp, MZ: marginal zone. Two-way analysis of variance with Dunnett's correction was used to analyze the data in (**C**) and (**E**). One-way analysis of variance with Dunnett's correction was used to analyze the data in Figure (**D**). Scale bar: 100 µm. ^*^P < 0.05; ^***^P < 0.001; ^****^P < 0.0001; ns, not significant.

As proof of concept, we intravenously injected PEGylated polystyrene nanoparticles into mice with different microbiota compositions (Figure 1A). We depleted the gut microbiota by treating the mice with an ABX cocktail administered in their drinking water for two weeks. To confirm the influence of gut microbiota influence on nanoparticles, we recolonized the ABX-treated mice by fecal microbial transplantation (FMT). We used normal mice and GF mice without treatments as controls. We used real-time quantitative polymerase chain reaction (RT-qPCR) to verify microbiota modulation by quantifying bacterial 16S rDNA content in the feces (Figure S1) 19. Commercially-available non-degradable polystyrene nanoparticles loaded with fluorescent dye were employed in this study. We coated the nanoparticles with polyethylene glycol (PEG) to increase their half-life and achieve a near-neutral zeta potential 20. The success of PEGylation was verified by a slightly larger hydrodynamic diameter with a near-neutral surface potential (Table S1).

After the establishment of the mouse model and PEGylation of the nanoparticles, we subsequently determined the biodistribution profile by intravenously injecting 100 nm PEGylated polystyrene nanoparticles into control, ABX, FMT and GF mice. We chose the 100 nm size because it is similar to the size used in FDA-approved nanoparticle-based therapeutics 21. Furthermore, nanoparticles with a diameter of 100 nm and below have been found to possess better penetration ability in solid tumors 22.

*Ex vivo* fluorescence imaging of the major organs showed a large nanoparticle accumulation in the lungs, liver and spleen (Figure 1B). Imaging of ABX and GF spleens and plasma revealed brighter fluorescence compared to that of control and FMT mice, suggesting a higher splenic accumulation and slower nanoparticle clearance. The absence of gut microbiota has been known to negatively impact the host immune response 23, which could potentially be the underlying reason behind the slower rate of clearance and higher splenic accumulation. To quantify the nanoparticle content accurately, nanoparticles were extracted and amounts were measured through HPLC (Figure S2A-E). The results were expressed as nanoparticle retention per gram of tissue, as a measure of the capability of certain organs to harbor nanoparticles, and as a percentage of injected dose (%ID) per gram of tissue, as a measure of the tendency of the nanoparticles to migrate to certain organs. Analysis of nanoparticle retention confirmed fluorescence imaging results, where ABX and GF spleens and plasma were found to contain higher nanoparticle retention and %ID/gram (Figure 1C-E).

To determine whether nanoparticle size affects the biodistribution profile, we used 20, 100 and 1000 nm nanoparticles. We also subjected the nanoparticles to PEGylation to minimize the large variation in surface potential. Size and zeta potential measurement showed that the PEGylation reduced this variation without considerably changing the size (Table S1). The results for all nanoparticle sizes showed higher nanoparticle retention and %ID/gram in ABX spleens (Figure S3).

We then used confocal laser scanning microscopy to assess the intrasplenic distribution profile. The results revealed that in control and FMT spleens, the nanoparticles mainly accumulated in the marginal zone. Interestingly, we found that in ABX and GF spleens, the nanoparticles were found not only in the marginal zone but also distributed throughout the red pulp (Figure 1F), indicating deeper penetration.

### Depletion of gut microbiota increases nanoparticle accumulation in tumors

**Figure 2:**
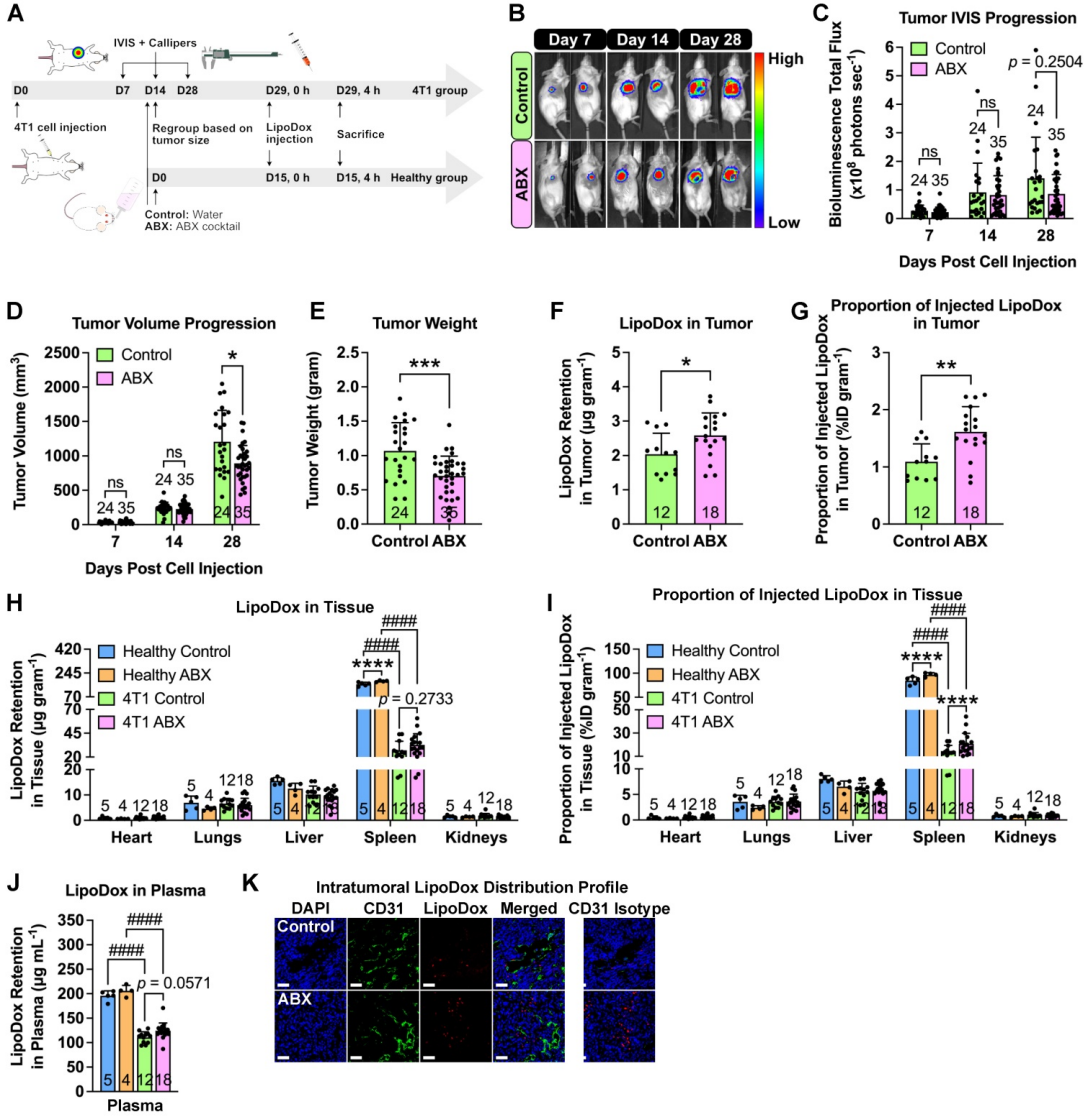
** Gut microbiota depletion increases LipoDox accumulation in TNBC tumors.** (**A**) Female BALB/c mice were injected with 10^4^ 4T1 cells to generate the 4T1 group. Both healthy (no tumor) and 4T1 group mice were subjected to gut microbiota modulation by ABX treatment two weeks after cell inoculation. At the end of the ABX treatment, the mice were injected with LipoDox (8 µg gram^-1^ body weight) *via* the tail vein. (**B**) Tumor progression was monitored through IVIS. (**C**) Quantification of bioluminescence total flux. (**D**) Tumor progression as monitored by volume measurement using calipers. (**E**) Tumor weight following excision. (**F-J**) Quantification of LipoDox accumulation in the tumor, major organs and plasma, expressed as (**F**) LipoDox content in the tumor, (**H**) each major organ and (**J**) plasma as well as (**G**) the proportion of LipoDox that went into the tumor and (**I**) each organ. (**K**) Representative confocal laser scanning microscopy images of intratumoral LipoDox distribution. Scale bar: 50 µm. Two-way analysis of variance with Sidak's correction was used to analyze the data in (**C**) and (**D**). Two-way analysis of variance with Tukey's correction was used to analyze the data in (**H**) and (**I**) One-way analysis of variance with Tukey's correction was used to analyze the data in (**J**). Two-tailed unpaired *t* test was used to analyze the data in (**E**), (**F**) and (**G**). ^*^ indicates significance between control and ABX, whilst ^#^ indicates significance between healthy and tumor-bearing groups. ^*^P < 0.05; ^**^P < 0.01; ^***^P < 0.001; ^****,####^*P* < 0.0001; ns, not significant.

To determine whether cancer nanomedicine is influenced by the gut microbiota, we injected female BALB/c mice with 4T1-Luc triple-negative breast cancer cells, depleted their microbiota by ABX treatment and administered liposomal doxorubicin (LipoDox) nanoparticles as nanotherapeutics (Figure 2A). We monitored the tumor growth by luciferase bioluminescence using the *in vivo* imaging system (IVIS) and tumor volume using digital calipers. Both measurements revealed that ABX tumors had smaller size at the end of the experiment (Figure 2B-D).

To determine the biodistribution profile, LipoDox injections were performed on healthy (no tumor) and tumor-bearing control and ABX mice. Tumor weight measurement confirmed that ABX tumors were smaller in size compared to the control (Figure 2E). A previous study showed that microbiota depletion by ABX treatment hindered the growth of several tumors, such as melanoma, and pancreatic and colon cancers and that the growth restriction was caused by the gut microbiota and not by ABX toxicity 1. Hence, we now confirm that similar growth impairment is also observed in breast cancer. We then extracted and measured the LipoDox content in all organs. To our surprise, we found significantly higher LipoDox retention and %ID/gram in ABX tumors compared to control tumors (Figure 2F-G). Furthermore, similar to our previous observation, for both healthy and tumor-bearing mice, we found an increase in LipoDox accumulation in ABX spleen and plasma (Figure 2H-J). We then assessed the intratumoral distribution profile by staining with CD31, an endothelial cell marker. Confocal laser scanning microscopy showed that LipoDox accumulated near the blood vessels in control tumors, while in ABX tumors, they accumulated farther away (Figure 2K).

In order to determine whether this phenomenon is also observable in other types of solid tumor, we injected male C57BL/6 mice with B16F10 melanoma cells, depleted their microbiota by ABX treatment and administered LipoDox nanoparticles as nanotherapeutics (Figure S4A). Similarly, we monitored the tumor growth using IVIS and tumor volume progression using digital calipers, both of which revealed that ABX tumors had smaller size at the end of the experiment (Figure S4B-D). This was also confirmed by tumor weight measurement (Figure S4E). LipoDox quantification in tumor revealed that ABX tumors had higher LipoDox retention (Figure S4F) and %ID/gram (Figure S4G) compared to control tumors. These results confirm that gut microbiota depletion can improve the accumulation of nanomedicine in the tumors.

### Increased LipoDox accumulation in the tumor leads to improved therapeutic efficacy

**Figure 3:**
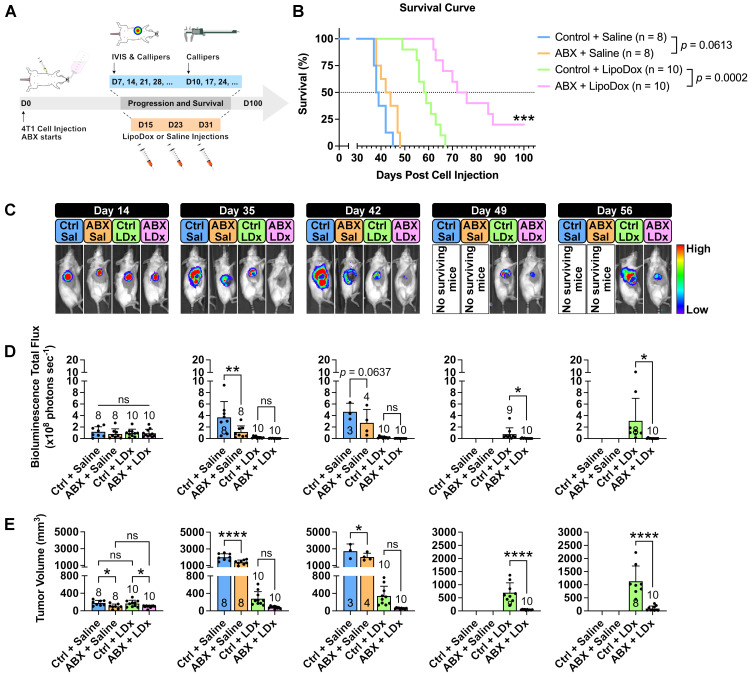
** Gut microbiota depletion increases the therapeutic efficacy of LipoDox treatment towards TNBC tumors and improves mouse survival.** (**A**) Female BALB/c mice were injected with 5 × 10^3^ 4T1 cells and were immediately grouped and subjected to gut microbiota modulation by ABX treatment, given three injections of LipoDox (8 µg gram^-1^ body weight) or saline *via* the tail vein and monitored until each mouse reached the endpoints. (**B**) Kaplan-Meier survival curve for all treatment groups. Pairs of survival curves were compared by log-rank (Mantel-Cox) test. (**C**) Weekly tumor growth was monitored through IVIS. Ctrl: control, Sal: saline, LDx: LipoDox. (**D**) Quantification of bioluminescence total flux. (**E**) Tumor progression as monitored by volume measurement using calipers. For both IVIS and tumor volume measurements, each piece of data from day 14 to day 42 was analyzed with one-way analysis of variance with Tukey's correction, while each piece of data from day 49 to day 56 was analyzed with two-tailed unpaired *t* test. Additionally, since n = 3 was used as a minimum, when the number of mice for a group dropped to below three over the course of the survival experiment period, that particular group was excluded from the statistical analysis. ^*^P < 0.05; ^**^P < 0.01; ^***^P < 0.001; ^****^P < 0.0001; ns, not significant.

We then sought to determine whether the increased accumulation in ABX tumors would influence LipoDox therapeutic efficacy. Three injections of LipoDox or saline were given to control and ABX mice weekly starting on D14 post cell inoculation. Tumor growth and mouse survival were subsequently monitored (Figure 3A). A Kaplan-Meier survival curve (Figure 3B) showed that microbiota depletion alone was not able to improve the median survival (control vs ABX = 38.5 vs 43 days, *p* = 0.0613). However, the combination of microbiota depletion and LipoDox injection was able to significantly improve the median survival (control vs ABX = 58.5 vs 74 days, *p* = 0.0002), with two of the mice surviving until the experimental time endpoint of 100 days.

Assessment of tumor growth by IVIS and volume (Figure 3C-E, Figure S5A-B) revealed smaller tumor size in ABX tumors, as expected (D14-42). Two weeks after the final injection of LipoDox/saline (D49), we observed a significant difference in tumor size between LipoDox-treated control and ABX mice; this indicated relapse for control + LipoDox mice but not for ABX + LipoDox mice. Three to four weeks after the final LipoDox injection (D56-63), ABX tumor size remained significantly smaller compared to control (Figure 3C-E, Figure S5A-B). During necropsy we observed lung metastasis, which could have contributed to death (Figure S5C). All of these results indicated that in the short term, LipoDox therapeutic efficacy was similar in both groups; however, over the long term, combination treatment of LipoDox and microbiota depletion managed to attenuate tumor growth with higher efficacy and suppress tumor relapse better. In the clinical setting, a long-term cancer suppression effect is desirable since this can prolong patient survival by preventing relapse.

### ABX treatment dramatically affects gut microbiota but not tumor microbiota

Several studies have shown that ABX treatment can alter bacterial population and diversity 1,24 and that the remaining bacteria after the treatment can exert their effects on their host. In order to identify the bacteria responsible for improved LipoDox therapeutic efficacy, DNA from aseptically-collected control and ABX stool samples were isolated and sent for bacterial 16S V3-V4 next generation sequencing. We found that after PCR amplification, only one out of 10 ABX-treated stool samples had enough bacterial DNA for sequencing. Following the sequencing, the actual abundance fecal bacterial DNA in ABX stool samples was much lower (~150 fold) compared to that of control (Figure S6A). This result confirmed the findings from our RT-qPCR analysis of stool samples that ABX treatment in this study depleted most of the gut microbiota (Figure S1). Relative abundance analysis showed that there was not much difference in bacterial population between control and ABX stools (Figure S6B).

We then turned our attention to possible changes in tumor microbiota caused by ABX treatment that could have affected LipoDox accumulation in the tumor. A recent study has shown that tumors contain their own distinct population of microbiota, with breast tumor microbiota in particular being the most diverse 25. DNA from aseptically-collected control and ABX breast tumor samples were isolated and sent for bacterial 16S V3-V4 next generation sequencing. Due to the low amount of bacterial DNA in tumors, a negative control was also included in the sequencing and used to subtract the results obtained for the tumor samples. Unlike fecal bacteria, ABX treatment did not cause changes in the abundance and diversity of tumor bacteria (Figure S7A-D). Taken together, these results indicated that changes in LipoDox accumulation in the tumor was caused by the depletion of gut microbiota, instead of the remaining gut microbiota after ABX treatment or tumor microbiota.

### Absence of gut microbiota increases spleen and tumor vascular permeability

**Figure 4:**
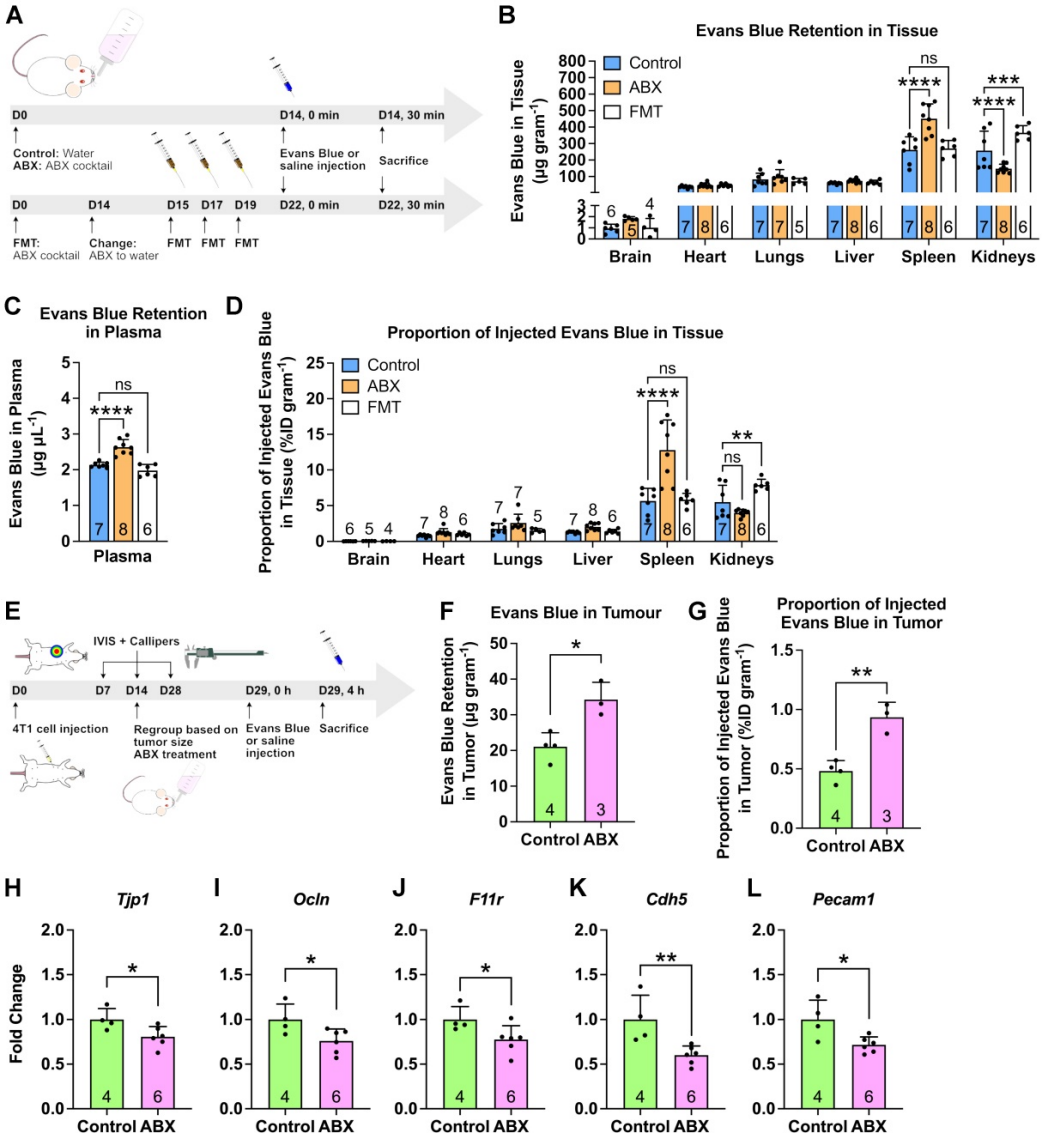
** Depletion of gut microbiota increased spleen and tumor vascular permeability.** (**A**) Mice were subjected to gut microbiota content modulation prior to systemic nanoparticle administration. Control and germ free mice were given normal drinking water. ABX mice were given ABX cocktail in the drinking water throughout the experiment. FMT mice were given ABX cocktail for two weeks followed by normal water and fecal transplant. At the end of the treatment, Evans Blue (4% w/v, 4 mg kg^-1^ body weight) were administered by intravenous injection *via* the tail vein. (**B-D**) Evans Blue assay to examine vascular permeability, expressed as dye content in (**B**) each organ and (**C**) plasma as well as (**D**) the proportion of injected dye. (**E**) Female BALB/c mice were injected with 10^4^ 4T1 cells and subjected to gut microbiota modulation by ABX treatment two weeks after cell inoculation. At the end of the ABX treatment, the mice were injected with Evans Blue (4% w/v, 4 mg kg^-1^ body weight) *via* the tail vein. (**F-G**) Evans Blue assay to examine tumor vascular permeability, expressed as (**F**) dye content in tumor and (**G**) the proportion of injected dye that went into the tumor. (**H-L**) Gene expression of vascular permeability regulators and mediators, (**H**) *Tjp1*, (**I**) *Ocln*, (**J**) *F11r*, (**K**) *Cdh5* and (**L**) *Pecam1* in the tumor following gut microbiota depletion. Data in (**B**) and (**D**) were analyzed with two-way analysis of variance with Tukey's correction. Data in (**C**) were analyzed with one-way analysis of variance with Tukey's correction. Data in (**F**) to (**L**) were analyzed with two-tailed unpaired *t* test. ^*^P < 0.05; ^**^P < 0.01; ^***^P < 0.001; ^****^P < 0.0001; ns, not significant.

A possible cause of higher nanoparticle accumulation is an increase in vascular permeability. The gut microbiota has previously been found to modulate the blood-brain barrier permeability 18. Since ABX treatment caused increased nanoparticle accumulation in the spleen of healthy male mice, we sought to examine the changes in splenic vascular permeability. We employed the Miles assay 26-28, which uses the Evans Blue dye (Figure S8A-B). Dye content quantification in the major organs following a systemic dye injection showed significantly increased dye retention and %ID/gram in ABX spleens, which returned to normal in FMT spleens, confirming our hypothesis (Figure 4A-D). We then further investigated whether increased tumor vascular permeability was the cause of the increased LipoDox accumulation by applying the Miles assay to tumor-bearing control and ABX female mice (Figure 4E). We found an increase in dye accumulation in ABX tumors, once again confirming our hypothesis (Figure 4F,G).

We then focused our attention on the changes in vascular permeability regulators (e.g. tight and adherens junctions) in the tumors 29-34. Using RT-qPCR, we examined the expression levels of *Tjp1*, *Ocln* and *F11r* genes, which encode for tight junction proteins ZO-1, occludin and junctional adhesion molecule-A, respectively. We also examined *Cdh5* (encodes for VE-cadherin) that makes up the adherens junction and *Pecam1* (encodes for CD31) an endothelial cell marker. We found that all of these genes were downregulated in the ABX tumor, indicating an increase in vascular permeability (Figure 4H-L). Therefore, we concluded that gut microbiota depletion induced an increase in vascular permeability, allowing more LipoDox to accumulate compared to that seen in the control tumor.

The gut microbiota has also been implicated in angiogenesis 16,17,35. Hence, we also investigated the effects of microbiota depletion on angiogenesis and tumor vasculature. We stained tumor sections with CD31 and used confocal laser scanning microscopy to capture whole-tumor images, which showed an obvious difference in blood vessel density (Figure S9A). Quantification of vascular density from these images revealed that ABX tumors had a lower vascular density (Figure S9B). We then investigated angiogenesis regulators that are also involved in vascular permeability regulation such as *Kdr* (encodes for VEGFR-2), angiopoietin-1 and -2 (*Angpt1* and *Angpt2*) and tyrosine kinase receptors *Tie1* (encodes for Tie-1) and *Tek* (encodes for Tie-2) 26,27,36-43. Overall, we observed downregulation of these markers in ABX tumors, indicating impaired angiogenesis and increased vascular permeability (Figure S9C-G). Analysis of tumor samples by transmission electron microscopy (TEM) did not reveal differences in the morphology of the blood vessels (Figure S9H). We then examined vessel maturation by assessing pericyte coverage through confocal laser scanning microscopy (Figure S9I-J). We found no alteration in pericyte coverage, indicating that maturation was not affected by gut microbiota depletion. Overall, our results showed that although microbiota depletion resulted in angiogenesis impairment, it did not affect the vasculatures themselves.

### Gut microbiota depletion and LipoDox treatment is safe

**Figure 5:**
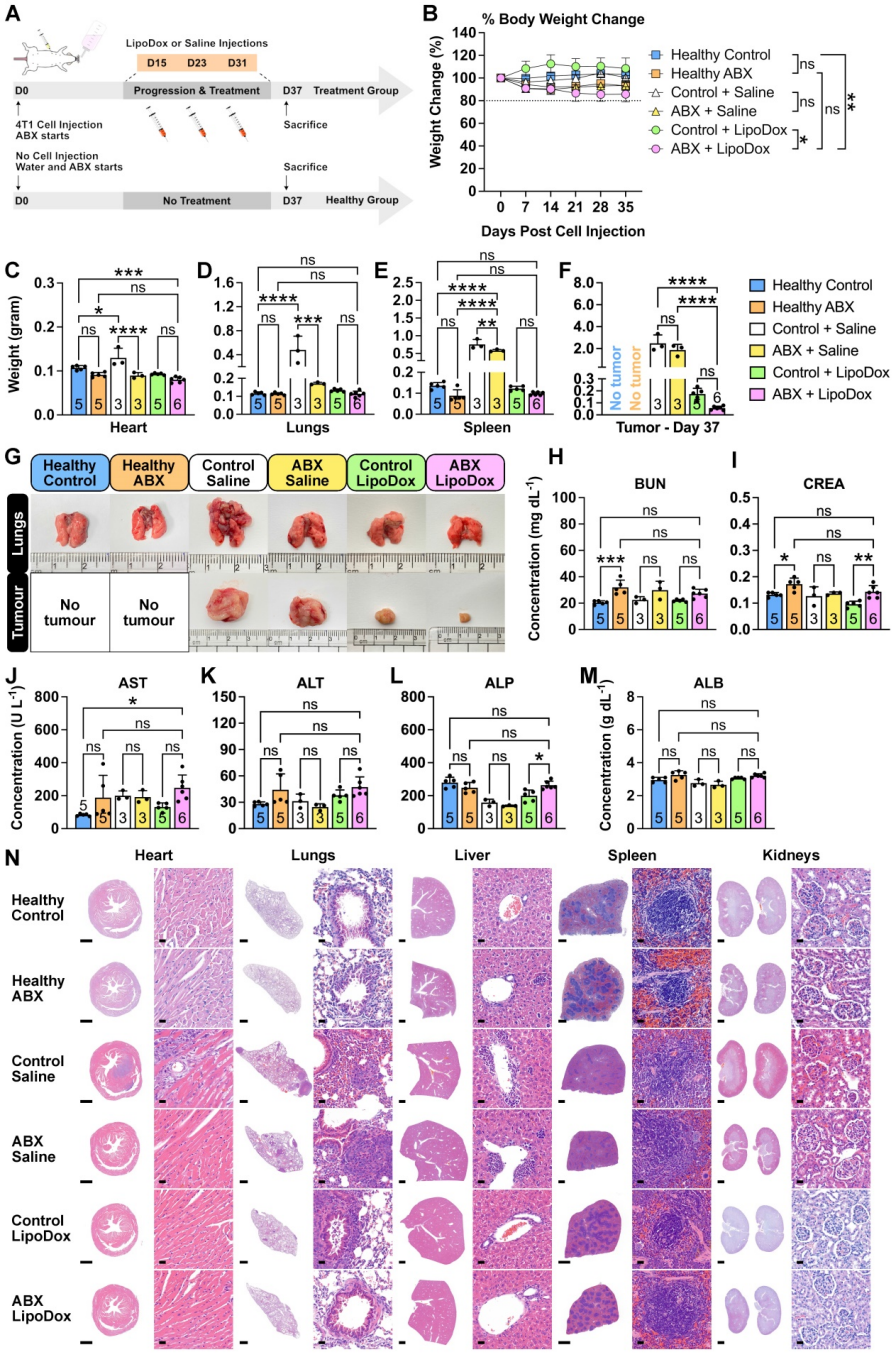
** Safety of gut microbiota modulation and LipoDox treatment.** (**A**) Female BALB/c mice were injected with 7.5 × 10^3^ 4T1 cells and were immediately grouped and subjected to gut microbiota modulation by ABX treatment, given three injections of LipoDox (8 µg gram^-1^ body weight) or saline *via* the tail vein and sacrificed on D37. (**B**) Change in body weight during the treatment. (**C-F**) Weight of the major organs: (**C**) heart, (**D**) lungs, (**E**) spleen and (**F**) tumor for each treatment group after sacrifice on D37. (**G**) Representative images of the lungs and tumors from each treatment group. (**H-M**) Serum biochemistry profile for kidney and liver function tests. Kidney function tests include: (**H**) blood urea nitrogen (BUN) and (**I**) creatinine (CREA) tests. Liver function tests include: (**J**) aspartate transaminase (AST), (**K**) alanine transaminase (ALT), (**L**) alkaline phosphatase (ALP) and (**M**) albumin (ALB) tests. (**N**) Histology analysis of major organs at the end of the treatment, analyzed by hematoxylin and eosin staining. Scale bar for whole organ images: 1 mm, for magnified images: 20 µm. Two-way analysis of variance with Tukey's correction was used to analyze the data in (**B**). Data in (**C**) to (**F**) and (**H**) to (**M**) were analyzed by one-way analysis of variance with Tukey's correction. ^*^P < 0.05; ^**^P < 0.01; ^***^P < 0.001; ^****^P < 0.0001; ns, not significant.

To investigate whether long-term ABX treatment and LipoDox injections cause any severe side effects, we subjected control and ABX tumor-bearing mice to saline and LipoDox treatments, in a similar manner to the survival experiment (Figure 5A). Healthy mice were used as biological control groups and were subjected to the same treatment as the tumor-bearing mice. Based on prior observations in the survival experiment, we wanted to ensure that for this group of mice, most or all of the injected LipoDox had been metabolized and that the saline-treated mice did not die due to the tumor. Hence, we sacrificed the mice a few days after the final saline/LipoDox injection (D37). At the end of the treatment, the body weight of ABX-treated mice was lower than that of controls. However, no ABX mice experienced more than 20% weight loss (Figure 5B). On D37, we sacrificed all the mice, collected their organs and recorded the weight (Figure 5C-F). We observed severe lung metastasis in the tumor-bearing control + saline group which also disseminated to the heart, resulting in heavier heart and lung weight (Figure 5C,D,G). We also observed splenomegaly in the saline-treated but not in the LipoDox-treated group (Figure 5E). Finally, we observed that on D37, the size of ABX + LipoDox-treated tumor was smaller than the control, although not statistically significant (Figure 5F,G). This was to be expected; similar to the results observed in the survival experiment, our treatment strategy resulted in better therapeutic efficacy in the long term.

To investigate whether the treatment incurred damage to the liver and kidneys, we performed serum biochemistry profiling (Figure 5H-M). In general, we saw an increase in several biochemical parameters in the ABX + LipoDox group, such as blood urea nitrogen (BUN, Figure 5H), aspartate transaminase (AST, Figure 5J) and alanine transaminase (ALT, Figure 5K); which can be ascribed to the use of ABX. Therefore, we concluded that the overall results indicated no liver and kidney damage from ABX + LipoDox treatment. We also performed a histology analysis to the organs through hematoxylin/eosin staining (Figure 5N). We confirmed the presence of metastasis in the control + saline heart, indicated by the dense population of hematoxylin-stained nuclei. Doxorubicin is known to be cardiotoxic, but histology analysis showed no cardiac damage. We also observed severe metastasis in the lungs in saline-treated groups as well as a few metastatic nodes in the lungs of control + LipoDox group. We did not observe any metastatic nodes in the lungs of ABX + LipoDox group. Furthermore, we were able to see some damage in the liver and spleen of saline-treated mice, but not of the LipoDox-treated mice. Finally, we did not observe any kidney damage. All assessments showed that ABX + LipoDox treatment was the safest; it did not adversely affect the organs and did not result in early lung metastasis.

### Cancer nanomedicine efficacy is improved by the absence, not the presence, of the gut microbiota

The rise of cancer immunotherapy suggests a new direction in cancer treatment; however, it is not without risks. Cancer immunotherapy using immune checkpoint blockers may result in unwanted immune-related adverse side effects 44. In such cases, cancer nanomedicine may be an alternative treatment. Here, we investigated the influence of the gut microbiota on cancer nanomedicine.

Our results showed that, surprisingly, ablation of gut microbiota resulted in augmented anti-tumor activity of nanoparticle-based therapy, strongly contrasting with findings for cancer immunotherapy to date 2-6. We believe the main reason for this discrepancy lies in the differing mechanisms of action through which these two therapeutic methods transpire. In cancer immunotherapy, the presence of the gut microbiota helps to prime the immune cells, increasing their anti-tumor activity and consequently augmenting the efficacy of immune checkpoint blockers 2-6. Conversely, nanoparticle-based treatments rely on nanoparticle accumulation in the tumor, which then relies on extravasation from the vasculature into the tumor. Our results showed that ablation of the gut microbiota resulted in an increase in vascular permeability, thereby facilitating nanoparticle entry into the tumor.

The results of our study imply that gut microbiota presence decreases tumor vascular permeability, which further implies that the presence of microbiota may decrease nanoparticle accumulation and that certain bacterial taxa may be responsible for vascular permeability regulation. The 16S next generation sequencing results further strengthen our findings that changes in vascular permeability is caused by the depletion of the gut microbiota and not by the remaining gut microbiota after ABX treatment or by the tumor microbiota. The main limitation of our study is that we did not identify the taxa responsible for vascular permeability regulation because we mainly focused on the impact of microbiota modulation on cancer nanomedicine. Prior to application in the clinic, future studies should first endeavor to identify the taxa responsible for vascular permeability modulation as well as the mechanism of action that underlies the communication between the gut microbiota and vascular permeability regulators and mediators. Our study also revealed that the microbiota modulation strategy can potentially be used to complement and improve existing nanoparticle-based strategies in cancer treatment and diagnosis. This strategy may also be used in cancer imaging by enhancing the ability of contrast dyes to accumulate in the tumor. This will be beneficial in improving diagnosis and increasing patients' chance of survival.

Lastly, we would like to note that although there are published studies concerning the relationship and interactions between the gut and the gut microbiota and nanomaterials, these studies focused specifically on orally-administered nanomaterials, such as those found in food packaging or additives 45,46. This route of administration enables the nanomaterials to directly interact with the gastrointestinal tract and the bacteria within; however, this route is not related to cancer nanomedicine since it relies on the intravenous administration of nanomaterials instead. Our study directly investigated the relationship between the gut microbiota and nanotherapeutics administered intravenously, which is the main route of therapeutics administration for cancer treatment.

## Conclusion

In summary, using a mouse model of triple-negative breast cancer, we have shown that ablation of the gut microbiota resulted in impaired tumor growth and increased tumor vascular permeability. Ablation of gut microbiota allowed more LipoDox nanoparticles to accumulate in the tumor, resulting in augmented long-term anti-tumor therapeutic efficacy and longer mouse survival in multiple mouse models. In the future, studies to determine the causal mechanism of action should be pursued, towards future translation in clinic. Furthermore, future studies should also endeavor to determine the bacterial taxa responsible for vascular permeability regulation. Manipulating the population of these taxa in conjunction with nanoparticle-based therapeutics or imaging agents may potentially be a novel therapeutic strategy against cancer. Additionally, the mechanism underlying the regulation of tumor vascular permeability and angiogenesis should also be thoroughly studied. We believe that our study is adaptable to existing cancer nanomedicine strategies and suggests a new direction in cancer therapy, imaging and diagnosis.

## Experimental Section/Methods

### Animal Experiments

For polystyrene nanoparticle biodistribution experiments, 11- to 14-week-old male WT BALB/c mice purchased from BioLasco were used. For breast cancer model and LipoDox biodistribution experiments, female 10- to 14-week-old WT BALB/c mice purchased from the National Laboratory Animal Center, Taiwan, were used. For GF mouse experiments, 14-week-old male BALB/c mice purchased from the National Laboratory Animal Center, Taiwan, were used. Mice were housed under a 12-hour day-night cycle with unlimited access to food and water. All animal experiments have been approved by Academia Sinica Institutional Animal Care and Use Committee (IACUC).

### 4T1 Triple-Negative Breast Cancer Model for Nanoparticle Biodistribution Study

Luciferase-expressing 4T1 (4T1-Luc) triple-negative breast cancer cells were cultured in Roswell Park Memorial Institute (RPMI) 1640 culture medium supplemented with 10% FBS, L-glutamine, sodium pyruvate and non-essential amino acids. For nanoparticle biodistribution study, 1 × 10^4^ cells suspended in 25 μL sterile PBS were subcutaneously injected into the mammary fat pad under the third mammary gland of 10 week old female BALB/c mice.

Tumor growth was monitored weekly through IVIS (PerkinElmer) starting from day 7 post injection. Mice were anesthetized through isoflurane inhalation and intraperitoneally injected with D-luciferin firefly potassium salt solution (200 μL, 15 mg mL^-1^ in PBS, Biosynth). A series of IVIS images was then captured (exposure time 30 sec, F/stop 4) until the bioluminescence intensity of each mouse reached the maxima. Tumor volume was measured weekly using calipers starting from day 7 post injection using the formula V = 0.5 × (W^2^ × L), where V is tumor volume, W is the tumor width and L is tumor length. Width and length are the smaller and larger perpendicular axes, respectively. Body weight was monitored weekly starting on the day of cell injection throughout the experiment.

The mice were regrouped into control and ABX groups at day 14 post cell inoculation (12 weeks old) and the ABX treatment was started on the same day. Mice were then injected with LipoDox and sacrificed at day 29 post cell injection (14 weeks old).

### Administration of PEG Polystyrene Nanoparticles, LipoDox and Evans Blue

Nanoparticles were administered when the male mice reached 14 weeks old. For the ABX group, this time point was at the end of the ABX treatment (day 14); whilst for the FMT group, this time point was at the end of the FMT treatment (day 7). Control and GF mice who did not receive any of these treatments were subjected to nanoparticle administration only after they had reached 14 weeks old. PEG polystyrene nanoparticles (40 μg gram^-1^ body weight) were administered by intravenous injection *via* the tail vein. Following the injection, the mice were left for 4 hours, anesthetized by Zoletil (100 μL through intraperitoneal injection) and sacrificed by perfusion with heparin (0.1%) in PBS. Tissue samples from the major organs (brain, heart, lungs, liver, spleen and kidneys) were collected, weighed and used for further analysis. Blood was collected through a cardiac puncture into a BD Vacutainer EDTA tube (BD Biosciences) and centrifuged (4 °C, 2,000 *g*) for 30 min to obtain plasma which was used for further analysis.

LipoDox were administered when the female mice reached 14 weeks old. For the healthy ABX group, this time point was at the end of the ABX treatment (day 15); whilst the healthy control group was subjected to LipoDox administration only after they had reached 14 weeks old. For the tumor-bearing control and ABX groups, this time point was at day 29 post cell inoculation, which also coincided with day 15 of the ABX treatment for the tumor-bearing ABX group. LipoDox (8 μg gram^-1^ body weight) was administered *via* the tail vein. Following the injection, the mice were left for 4 hours, anesthetized by Zoletil (100 μL through intraperitoneal injection) and sacrificed by perfusion with heparin (0.1%) in PBS. Tissue samples from the major organs (brain, heart, lungs, liver, spleen and kidneys) and tumor samples from tumor-bearing mice were collected, weighed and used for further analysis. Blood was collected through a cardiac puncture into a BD Vacutainer EDTA tube (BD Biosciences) and centrifuged (4 °C, 2,000 *g*) for 30 min to obtain plasma used for further analysis. GF mice were also subjected to the same treatment.

For vascular permeability assay, Evans Blue was administered at the same time point explained above for healthy male mice (14 weeks old) and tumor-bearing female mice (14 weeks old). Evans Blue (4% w/v, 4 mg kg^-1^ in 0.9% sterile saline) or 0.9% sterile saline was intravenously injected *via* the tail vein. The mice were left for 30 min and sacrificed following the procedure described above. Tissue samples from the major organs (brain, heart, lungs, liver, spleen and kidneys) and tumor samples from tumor-bearing mice were collected, weighed and used for further analysis. Blood was collected through a cardiac puncture into a BD Vacutainer EDTA tube (BD Biosciences) and centrifuged (4 °C, 2,000 *g*) for 30 min to obtain plasma which was used for further analysis.

### Nanoparticle and Evans Blue Extraction from Biological Samples

#### Extraction of Yellow-Green Fluorescent Dye from Biological Samples

Tissue samples (approx. 100 mg) were weighed; and zirconia beads and ddH_2_O (500 μL) were added to aid the homogenization process. Samples were thoroughly homogenized using a Roche MagNA Lyser instrument in 30 secs bursts (maximum speed 7,000) for a total of 2.5 min (5 bursts). Plasma (100 μL) was taken, diluted with ddH_2_O (final volume 500 μL) and mixed thoroughly. *O*-xylene (300-500 μL) was added into the homogenized tissue suspension or plasma samples, and the mixture was then subjected to vigorous mixing by vortex followed by sonication. The sonication was performed for 10 min in a series of 2 min bursts with vigorous mixing in between. The mixture was then frozen at -80 °C for 30 min, thawed at room temperature and centrifuged at 14,000 rpm (4 °C, approx. 18,800 *g*) for 30 min. The organic layer (100 μL) was then taken for HPLC analysis using Waters e2695 separation module equipped with Waters 2475 FLR Detector and Waters X-Bridge C18 column (4.6 × 250 mm, 5 μm). The mobile phase used for the quantification of yellow-green polystyrene nanoparticles consisted of methanol (77%) and water (23%) with isocratic elution (0.8 mL min^-1^). The fluorescence detector wavelength was set to 505 nm for excitation and 515 nm for emission. The temperature of the column was set to 40 °C throughout the experiment. The area under the curve of the fluorescent dye peak (*t*_r_ ~9.3 min) was quantified.

#### Extraction of Doxorubicin from Biological Samples

Tissue samples (approx.100 mg) were weighed; and zirconia beads, cell lysis buffer (1,000 μL) containing sucrose (0.25 M), tris-HCl (5 mM), MgSO_4_ (1 mM), CaCl_2_ (1 mM) and adjusted to pH 6.7, and triton X-100 solution (100 μL, 10% v/v) were added to aid the homogenization process. Samples were thoroughly homogenized using a Roche MagNA Lyser instrument in 30 s bursts (maximum speed 7,000) for a total of 2.5 min (5 bursts). Plasma (100 μL) was taken, diluted with the cell lysis buffer (1,000 μL) and triton X-100 (100 μL, 10% v/v) solution and mixed thoroughly. The homogenized tissue suspension or plasma samples were then sonicated for 30 min (3 × 10 min with vigorous mixing in between). An amount (200-600 μL) was placed into a new tube containing acidified alcohol (600-1,000 μL, 0.75 *N* HCl in 70% EtOH) and mixed thoroughly. The suspension was left at -20 °C overnight, thawed at room temperature and centrifuged (4 °C, 10,000 *g*) for 30 min the following day. The supernatant (100 μL) was then taken for HPLC analysis using a Waters e2695 separation module equipped with Waters 2475 FLR Detector and a Waters X-Bridge C18 column (4.6 × 250 mm, 5 μm). The mobile phase used for the quantification of doxorubicin consisted of 0.1% HCOOH in water and 0.1% HCOOH in acetonitrile (ACN). A gradient elution (1.0 mL min^-1^) was used, with an initial water/ACN ratio of 95:5 which was changed linearly to 35:65 over 20 min, held for 5 min and decreased back at 25 min to 95:5 until the end of the 40 min analysis. The fluorescence detector wavelength was set to 480 nm for excitation and 600 nm for emission. The temperature of the column was set to 40 °C throughout the experiment. The area under the curve of the doxorubicin peak (*t*_r_ ~12.7 min) was quantified.

#### Extraction of Evans Blue from Biological Samples

Tissues (30-100 mg) were weighed; and zirconia beads and DMF (500 μL) were added to aid the homogenization process. Samples were thoroughly homogenized using a Roche MagNA Lyser instrument in 30 s bursts (maximum speed 7,000) for a total of 2.5 min (5 bursts). Plasma (8 μL) was added into a tube, and DMF (500 μL) was added and mixed thoroughly. Evans Blue dye was extracted by heating the mixtures at 60 °C overnight, followed by centrifugation (21,000 *g*) for 15 minutes the following day. The supernatant (200 μL) was taken into a 96-well plate and its absorbance was measured using a UV/Vis spectrometer (SpectraMax190 Microplate Reader) at 620 nm with background correction at 740 nm. Tissue and plasma samples from mice injected with 0.9% saline were subjected to similar treatment and used as blanks.

### Therapeutic Efficacy and Survival Study Procedure

For this study, 5 × 10^3^ 4T1 cells suspended in 25 μL sterile PBS were subcutaneously injected into the mammary fat pad under the third mammary gland of female BALB/c mice. The mice were immediately separated into control and ABX groups and ABX treatment was started on the same day. Tumor growth and volume were monitored once and twice weekly using IVIS and calipers, respectively, starting from day 7 post injection following the aforementioned protocol. Body weight was monitored weekly throughout the experiment starting on the day of cell injection. The mice were treated with three intravenous injections of either 0.9% sterile saline or LipoDox (8 μg gram^-1^ body weight) *via* the tail vein on days 15, 23 and 31. Mice were sacrificed when the tumor length reached 20 mm, when there was a sudden and sharp decrease in body weight, when the mice were weak and unable to move, when they experienced difficulty breathing or when 100 days post cell injection was reached.

### Software

Size and zeta potential measurement was performed and processed using Malvern Zetasizer software (version 7.11). IVIS images of tumor bioluminescence and fluorescence imaging of nanoparticles in tissue were collected and processed using Living Image 3.1 or 3.2 software. HPLC analysis was performed and processed using Waters Empower 3 software. Real-Time qPCR data was collected and processed using Applied Biosystem 7500 Real-Time qPCR software. Confocal microscopy images were taken and processed using ZEISS ZEN Black software. Hematoxylin and eosin-stained histology images were collected using 3D Histech Pannoramic Scanner software and processed using 3D Histech CaseViewer software. Image quantification was performed using FIJI (ImageJ). Figures were assembled in Affinity Designer (version 1.8.3, MacOS).

### Statistics

GraphPad Prism 9 (version 9.2.0) for MacOS was used for all statistical analysis and graph plotting. For all statistical analyses, n = 3 was used as a minimum to obtain statistically meaningful and significant results. A *P* value of less than 0.05 was considered significant. For *in vivo* tumor growth and LipoDox biodistribution in tumor-bearing mice, at least three independent experiments were performed. Mice were divided into groups randomly. For breast cancer groups, the day when ABX treatment started, the mice were divided into control and ABX groups in a way so that there was no statistical significant difference (in IVIS bioluminescence and tumor volume) between the two groups. Statistical methods used for the data presented in the main figures are as follows.

## Supplementary Material

Supplementary method, figures and tables.

## Abbreviations

ABX: antibiotics
ALB: albumin
ALP: alkaline phosphatase
ALT: alanine transaminase
AST: aspartate transaminase
BUN: blood urea nitrogen
CREA: creatinine
FMT: fecal microbial transplantation
GF: germ free
HPLC: high performance liquid chromatography
ID: injected dose
IVIS: *in vivo* imaging system
LipoDox and LDx: liposomal doxorubicin
PEG: polyethylene glycol
RT-qPCR: real-time quantitative polymerase chain reaction
TEM: transmission electron microscopy
