# Supplementary material for: Depletion of gut microbiota improves the therapeutic efficacy of cancer nanomedicine
Source: Theranostics. 2022 Oct 17;12(17):7390–403. doi: 10.7150/thno.73873 (PMC9691371; doi:10.7150/thno.73873)
Supplement: Supplementary file 1 — Supplementary method, figures and tables. [file thnov12p7390s1.pdf]

Supporting Information

Depletion of gut microbiota improves the therapeutic efficacy of cancer nanomedicine

Ray Putra Prajnamitra[1], Yuan-Yuan Cheng[1], Chaw Yee Beh[1], Chien-Yi Lu[1], Jen-Hao Lin[1], Shu-Chian Ruan[1], Sheng-Lun Chen[1], Hung-Chih Chen[1], Ruey-Bing Yang[1], Patrick Ching-Ho Hsieh[1,2,*]

1. Institute of Biomedical Sciences, Academia Sinica, Taipei 115, Taiwan
2. Institute of Medical Genomics and Proteomics and Institute of Clinical Medicine, National Taiwan University College of Medicine, Taipei 100, Taiwan

*Corresponding author, E-mail: phsieh@ibms.sinica.edu.tw

SUPPLEMENTARY METHODS

Antibiotics Treatment

An antibiotics (ABX) cocktail containing ampicillin (Sigma, 250 mg L^{-1}), metronidazole (Sigma, 250 mg L^{-1}), neomycin (Sigma, 250 mg L^{-1}) and vancomycin (Sigma, 125 mg L^{-1}) was administered to mice *via* drinking water for 14 days starting when the mice reached 11 weeks of age for the FMT group or 12 weeks of age for the ABX group. For 4T1 tumor-bearing mice, the ABX treatment was given for 15 days, starting at day 14 post cell inoculation when the mice were 12 weeks old. The ABX was changed every two days to ensure freshness and cages were changed daily to ensure sterility.

Fecal Microbiome Transplantation (FMT) Procedure

Freshly collected stool pellets (150 mg) from non-ABX-treated, age- and gender-matched control mice were cut into small pieces and suspended in 3 mL freshly-sterilized PBS. The suspension was vigorously vortexed and spun down. The supernatant was collected and fecal solution (200 µL) was carefully administered to each mouse *via* oral gavage using polypropylene feeding tubes (Instech Lab). At the end of the 14-day ABX treatment, mice were given normal drinking water for one day (assigned as day 0) and the transplant was performed on days 1, 3 and 5.

16S rDNA Quantification

The absence of gut microbiota in germ free mice, depletion of the gut microbiota by ABX treatment and recolonization by FMT were all confirmed through 16S rDNA quantification. DNA was extracted from stool samples (approx. 100 mg) using an InnuPREP Stool DNA Kit (Analytik Jena) or EasyPrep Stool Genomic DNA Kit (Biotools, Taiwan) following the manufacturer's instructions. The isolated DNA was then subjected to RT-qPCR analysis to quantify changes in bacterial load in the samples.

Subcutaneous B16F10 Melanoma Model for Nanoparticle Biodistribution Study

Luciferase- and GFP-expressing B16F10 (B16F10-Luc-GFP) melanoma cells were cultured in

Dulbecco's Modified Eagle Medium (DMEM) high glucose medium supplemented with 10% FBS. On the day of inoculation, 5×10^4 cells suspended in 25 µL sterile PBS were subcutaneously injected into the right flank of 10 week old male C57BL/6 mice.

Tumor growth was monitored weekly through IVIS (PerkinElmer) starting from day 7 post injection. Mice were anesthetized through isoflurane inhalation and intraperitoneally injected with D-luciferin firefly potassium salt solution (200 µL, 15 mg mL^{-1} in PBS, Biosynth). A series of IVIS images was then captured (exposure time 60 sec, F/stop 1) until the bioluminescence intensity of each mouse reached the maxima. Tumor volume was measured weekly using calipers starting from day 14 post injection using the formula $V = 0.5 \times (W^2 \times L)$, where V is tumor volume, W is the tumor width and L is tumor length. Width and length are the smaller and larger perpendicular axes, respectively. Body weight was monitored weekly starting on the day of cell injection throughout the experiment.

The mice were regrouped into control and ABX groups 7 days post cell inoculation and the ABX treatment was started on the same day. Mice were then injected with LipoDox and sacrificed at day 22 post cell injection.

Construction of Polystyrene Nanoparticle Standard Curves

Extraction of Yellow-Green Fluorescent Dye from Nanoparticles

For each of the nanoparticle sizes, standards were diluted in ddH_2O into a series of smaller concentrations ranging from 0.01-1.00 µg mL^{-1}. Into these standard solutions, *o*-xylene (300 µL) was added and the mixture was then subjected to vigorous mixing by vortex followed by sonication. The sonication was performed for 10 min in a series of 2 min bursts with vigorous mixing in between. The mixture was then frozen at –80 °C for 30 min, thawed at room temperature and centrifuged at 14,000 rpm (4 °C, approx. 18,800 *g*) for 30 min. The organic layer (100 µL) was then taken for HPLC analysis and standard curves were constructed.

HPLC Quantification of Yellow-Green Fluorescent Dye from Nanoparticles

The HPLC analysis for all nanoparticle sizes was performed using Waters e2695 separation module equipped with Waters 2475 FLR Detector and Waters X-Bridge C18 column (4.6 x 250 mm, 5 µm). The mobile phase used for the quantification of yellow-green polystyrene nanoparticles consisted of methanol (77%) and water (23%) with isocratic elution (0.8 mL min^{-1}). The fluorescence detector wavelength was set to 505 nm for excitation and 515 nm for emission. The temperature of the column was set to 40 °C throughout the experiment. The area under the curve of the fluorescent dye peak (t_r ~9.3 min), which represents the dye concentration in the solution, was quantified.

Construction of LipoDox Standard Curves

Extraction of Doxorubicin from LipoDox

Cell lysis buffer containing sucrose (0.25 M), tris-HCl (5 mM), $MgSO_4$ (1 mM) and $CaCl_2$ (1 mM) was prepared and adjusted to pH 6.7. LipoDox (2% w/v, TTY Biopharm, Taiwan) was diluted in ddH_2O into a series of smaller concentrations ranging from 0.0083-8.3333 µg mL^{-1}. Cell lysis buffer (1,000 µL) and triton X-100 (100 µL, 10% v/v) solution were added to these standard solutions (100 µL) and sonicated for 30 min (3x 10 min with vigorous mixing in between). The mixtures (200 µL) were put into new tubes containing acidified alcohol (1,000 µL, 0.75 *N* HCl in 70% EtOH) and mixed thoroughly. The suspension was left at –20 °C overnight, thawed at room temperature and centrifuged (4 °C, 10,000 *g*) for 30 min the following day. The supernatant (100 µL) was taken for HPLC analysis and standard curves were constructed.

HPLC Quantification of LipoDox

The HPLC analysis for all nanoparticle sizes was performed using Waters e2695 separation module equipped with Waters 2475 FLR Detector and Waters X-Bridge C18 column (4.6 x 250 mm, 5 µm). The mobile phase used for the quantification of doxorubicin consisted of 0.1% HCOOH in water and

0.1% HCOOH in acetonitrile (ACN). A gradient elution (1.0 mL min^{-1}) was used, with an initial ratio of water/ACN of 95:5 which was changed linearly to 35:65 over 20 min, held for 5 min and decreased back at 25 min to 95:5 until the end of the 40 min analysis. The fluorescence detector wavelength was set to 480 nm for excitation and 600 nm for emission. The temperature of the column was set at 40 °C throughout the experiment. The area under the curve of the doxorubicin peak (t_r ~12.7 min), which represents doxorubicin concentration in the solution, was quantified.

Construction of Evans Blue Standard Curves

Extraction of Evans Blue

Evans Blue (Sigma) was dissolved in 0.9% sterile saline, diluted into a series of smaller concentrations ranging from 0.3-70.0 µg mL^{-1} and put in Eppendorf tubes. *N*,*N*-dimethylformamide (DMF, 500 µL) was added to these standard solutions (100 µL) and mixed thoroughly. Evans Blue dye was extracted by heating the mixture at 60 °C overnight and the mixture was centrifuged (4 °C, 21,000 *g*) for 15 mins the following day. The supernatant (200 µL) was quantified using UV/Vis spectroscopy.

UV/Vis Spectroscopy for Evans Blue Quantification

Following the extraction and centrifugation detailed above, the supernatant was taken and added into a 96-well plate and its absorbance was measured using a UV/Vis spectrometer (SpectraMax190 Microplate Reader) at 620 nm with background correction at 740 nm. A mixture of 0.9% saline in DMF was used as a blank. The results were linear up to 70 µg mL^{-1}.

Generation and Characterization of PEG Polystyrene Nanoparticles

PEGylation of Polystyrene Nanoparticles

An Amicon Ultra Centrifugal Filter tube (MWCO 30 kDa) was rinsed twice with ddH_2O (spun at 2,300 *g*, 4 °C). Immediately afterwards, fluorescent yellow-green polystyrene nanoparticles [500 µL,

Invitrogen, Cat. No. F8787 (20 nm), F8803 (100 nm) and F8823 (1,000 nm)] were added and centrifuged (2,300 *g*, 4 °C). The nanoparticles were then resuspended in MES buffer (500 μL, 50 mM, pH 6.0). MeO-PEG-NH_2 (60 mg, MW 5000, Nanocs, Taiwan), sulfo-NHS (Sigma, 14 mg) and EDC solution (200 μL, 20 mg mL^{-1} in 50 mM MES pH 6.0, Sigma) were added into the nanoparticle suspension and the mixture was stirred in the dark for 30 min. Borate buffer pH 8.2 (50 μL) was then added and the pH was adjusted to 7.00 ± 0.03 with 1 *N* NaOH and the resulting mixture was stirred in the dark overnight at room temperature.

Purification of PEG Polystyrene Nanoparticles

An Amicon Ultra Centrifugal Filter tube (MWCO 30 kDa) was rinsed twice with ddH_2O (spun at 2,300 *g*, 4 °C). The reaction mixture was added to the filter and spun (2,300 *g*, 4 °C) until most of the liquid was filtered out. The nanoparticle-containing supernatant was then washed three times with ddH_2O, followed by centrifugation to remove the remaining buffer. The nanoparticles were then re-suspended in ddH_2O (400 μL) and stored at 4 °C in the dark. The success of PEGylation was confirmed through size and zeta potential measurements and the concentration of the PEGylated polystyrene nanoparticles was determined through HPLC analysis.

Size and Zeta Potential Measurements of PEG Polystyrene Nanoparticles

PEG polystyrene nanoparticles or LipoDox nanoparticles (1 μL) were diluted 1,000x in a 0.9% sterile saline solution and subjected to size and zeta potential measurement using a Malvern Zetasizer Nano ZS instrument.

Quantification of PEG Polystyrene Nanoparticles

Following confirmation of PEGylation success through size and zeta potential measurements, the PEGylated polystyrene nanoparticles were diluted 100,000x in ddH_2O. The diluted samples were then

extracted following the procedure described above and subjected to HPLC analysis to quantify the concentration of PEGylated nanoparticles.

Therapeutic Efficacy Study and Toxicity Analysis Procedure

For the therapeutic efficacy study, 7.5×10^3 cells suspended in 25 μL sterile PBS were subcutaneously injected into the mammary fat pad under the third mammary gland of female BALB/c mice. The mice were immediately separated into control and ABX groups and ABX treatment was started on the same day. Tumor growth and volume were monitored weekly through IVIS and calipers starting from day 7 post injection following the aforementioned protocol. Body weight was monitored weekly starting on the day of cell injection throughout the experiment. The mice were treated with three intravenous injections of LipoDox (8 μg $gram^{-1}$ body weight) *via* the tail vein on days 15, 22 and 28. One week after the final injection, the mice were sacrificed. Tissue samples (brain, heart, lungs, liver, spleen, kidneys and tumor) were collected, weighed and fixed in PFA (4%) further analysis. Blood was collected through a cardiac puncture, left to clot and centrifuged (4 °C, 2,000 *g*) for 30 min to obtain the serum used for liver and kidney function tests.

Immunofluorescence Staining

<u>Spleen: Intrasplenic Polystyrene Nanoparticle Distribution Profile</u>

Spleen samples were fixed in paraformaldehyde (4%), dehydrated in sucrose (15% and 30% successively), embedded and frozen in OCT medium at –80 °C. Frozen tissue samples were cryosectioned to 10 μm thickness and mounted on a slide. The sections were washed with PBS and the nuclei were stained with DAPI (1:1,000).

<u>Tumor: Intratumoral LipoDox Distribution Profile and Vascular Density (CD31 Staining)</u>

Fresh tumor samples from LipoDox-treated mice were immediately embedded in OCT, frozen and cryosectioned in a 10 μm thickness and mounted on a slide. The sections were then washed with PBS

and blocked with PBST (0.1% Tween-20 in PBS) containing FBS (10%) and goat serum (10%). To visualize the blood vessels, the sections were stained with rat anti-mouse CD31 (1:100, clone MEC 13.3, BD Biosciences) blocking buffer overnight at 4 ºC. Isotype anti-IgG2a κ (1:100, clone R35 95, BD Biosciences) was used as a negative control. Following three rounds of washing, the sections were stained with goat anti-rat IgG AF488 secondary antibody (1:200, Invitrogen) for 1 h at room temperature and the nuclei were counterstained with DAPI (1:1,000) for 5 min at room temperature.

Tumor: Pericyte Coverage (CD31 and α-SMA Staining)

Fresh tumor samples were immediately embedded in OCT, frozen and cryosectioned in a 10 µm thickness and mounted on a slide. The sections were subjected to CD31 staining as described above. Following three rounds of wash, the sections were stained with goat anti-rat IgG AF488 secondary antibody (1:200, Invitrogen) and anti-αSMA Cy3 (1:250, clone 1A4, Sigma) for 1 h at room temperature and the nuclei were counterstained with DAPI (1:1,000) for 5 min at room temperature.

16S V3-V4 Next Generation Sequencing

Fecal and tumor samples were collected aseptically inside a sterilized hood. DNA samples were then extracted using EasyPrep Genomic DNA kit (BioTools, Taiwan) according to the manufacturer's instructions. DNA samples were then sent to BioTools Taiwan for sequencing analysis. The sequence results were then processed using QIIME 2 (version 2020.11). Primer-trimmed sequences were clustered using the q2-dada2 plugin. Phylogenetic information was obtained through Silva database v.138. Analysis of bacterial abundance and taxonomic diversity were performed with MicrobiomeAnalyst (https://www.microbiomeanalyst.ca/).

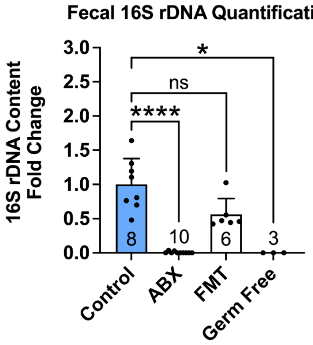

Figure S1. Mouse gut microbiota profile after various treatments. Two weeks of ABX treatment successfully depleted the gut microbiota from the mice and FMT treatment restored the microbiota population. Control and germ-free mice were used as positive and negative controls, respectively. The data was analyzed with Kruskal-Wallis test with Dunn's correction, $^{*}P < 0.05$; $^{****}P < 0.0001$; ns, not significant.

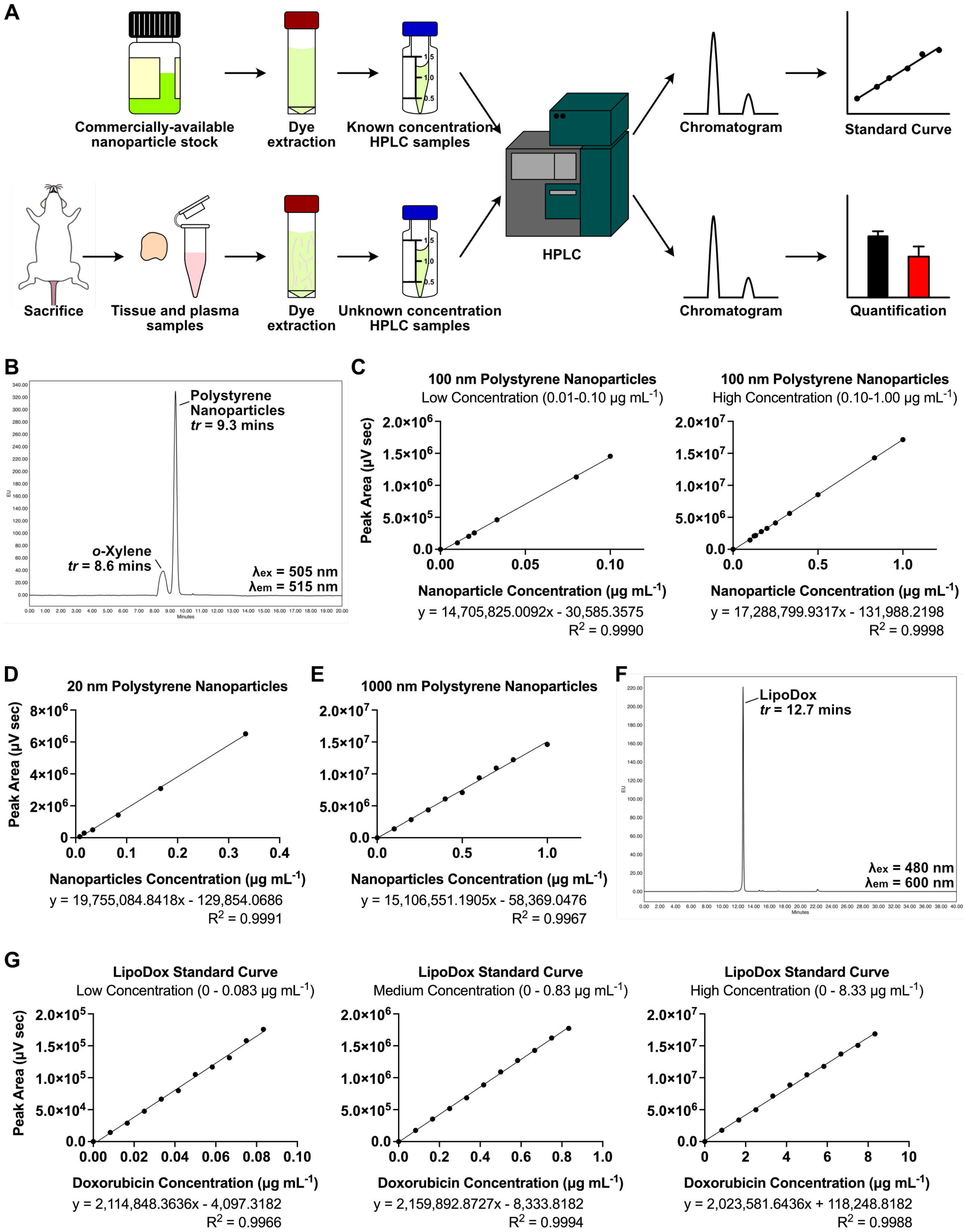

Figure S2. Standard curves of various nanoparticles. (**A**) A serial dilution of stock polystyrene nanoparticles was prepared and their fluorescence intensity was measured by HPLC to construct standard curves which were then used to quantify nanoparticle content in tissue and plasma samples.

(**B**) Representative polystyrene nanoparticle chromatogram. (**C-E**) Standard curves for different-sized polystyrene nanoparticles used in this study: (**C**) 100 nm for experiments with control, ABX, FMT and germ-free mice; (**D**) 20 nm and (**E**) 1000 nm for size dependency experiments. (**F**) Representative LipoDox chromatogram. (**G**) Standard curves for LipoDox nanoparticles.

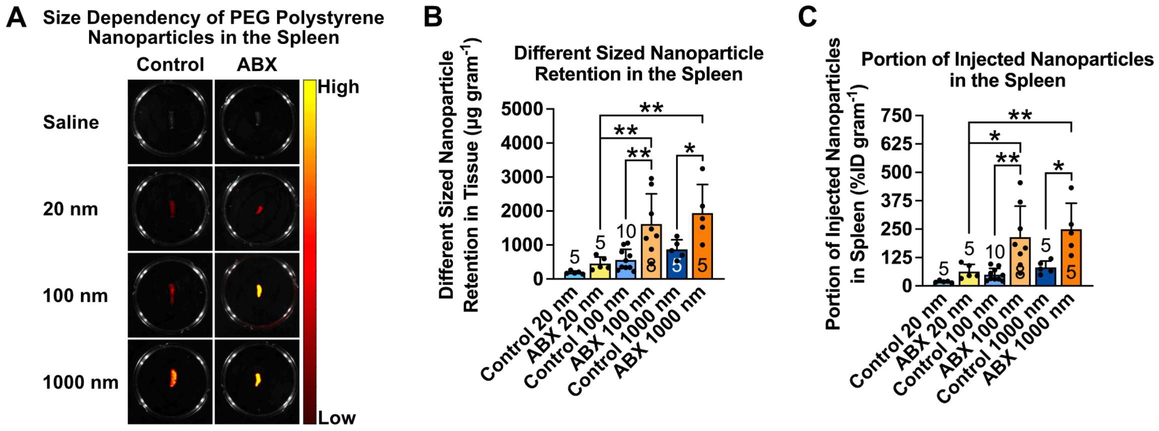

Figure S3. Biodistribution of different-sized polystyrene nanoparticles in the spleen following gut microbiota depletion. (**A**) Representative fluorescence images of spleen samples harboring different-sized polystyrene nanoparticles. (**B**) Nanoparticle retention in the spleen. (**C**) Proportion of injected nanoparticles that entered the spleen. Both were analyzed with one-way analysis of variance with Tukey's correction. $^{*}P < 0.05$; $^{**}P < 0.01$.

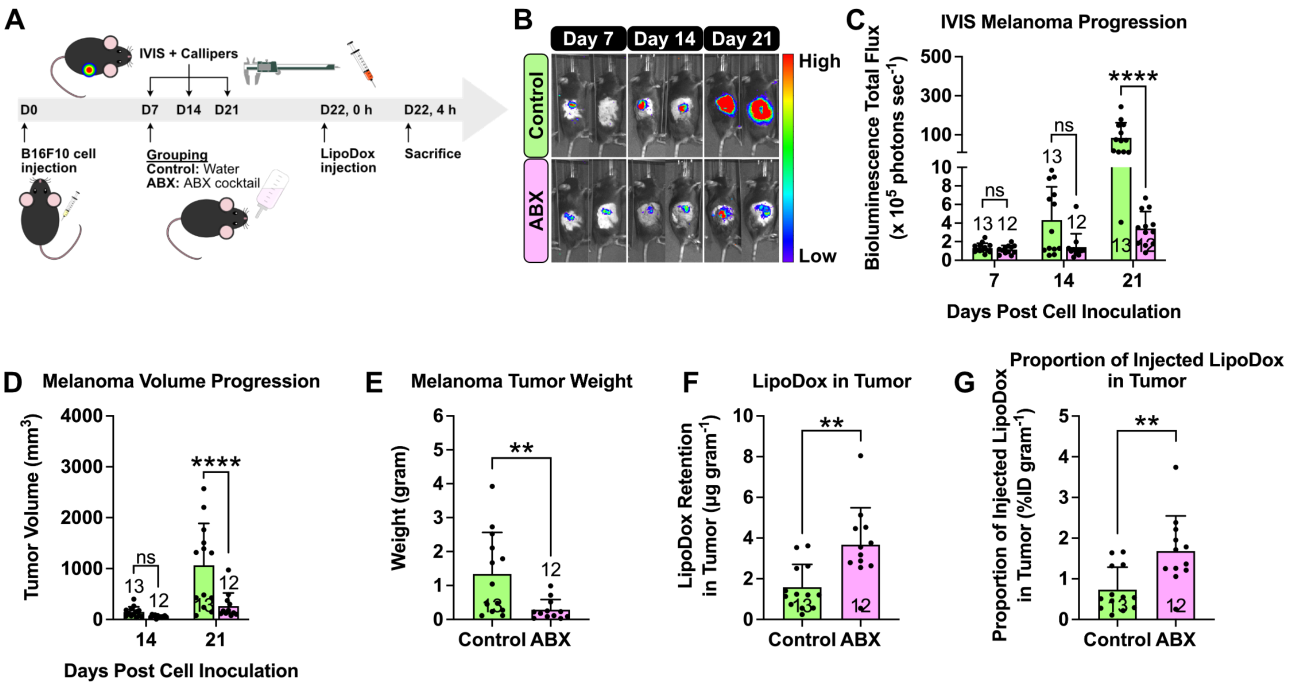

Figure S4. Gut microbiota depletion increases LipoDox accumulation in melanoma tumors. (**A**) Male C57BL/6J mice were injected with 5 × 10^4 B16F10 melanoma cells and subjected to gut microbiota modulation by ABX treatment one week after cell inoculation. At the end of the ABX treatment, the mice were injected with LipoDox (8 µg $gram^{-1}$ body weight) *via* the tail vein. (**B**) Tumor progression was monitored through IVIS. (**C**) Quantification of bioluminescence total flux. (**D**) Tumor progression as monitored by volume measurement using calipers. (**E**) Tumor weight following excision. (**F-G**) Quantification of LipoDox accumulation in the tumor, expressed as (**F**) LipoDox content in the tumors and (**G**) the proportion of injected LipoDox that went into the tumors. Two-way analysis of variance with Sidak's correction was used to analyze the data in (**C**) and (**D**). Two-tailed unpaired *t* test was used to analyze the data in (**E**), (**F**) and (**G**). $^{**}P < 0.01$; $^{****}P < 0.0001$; ns, not significant.

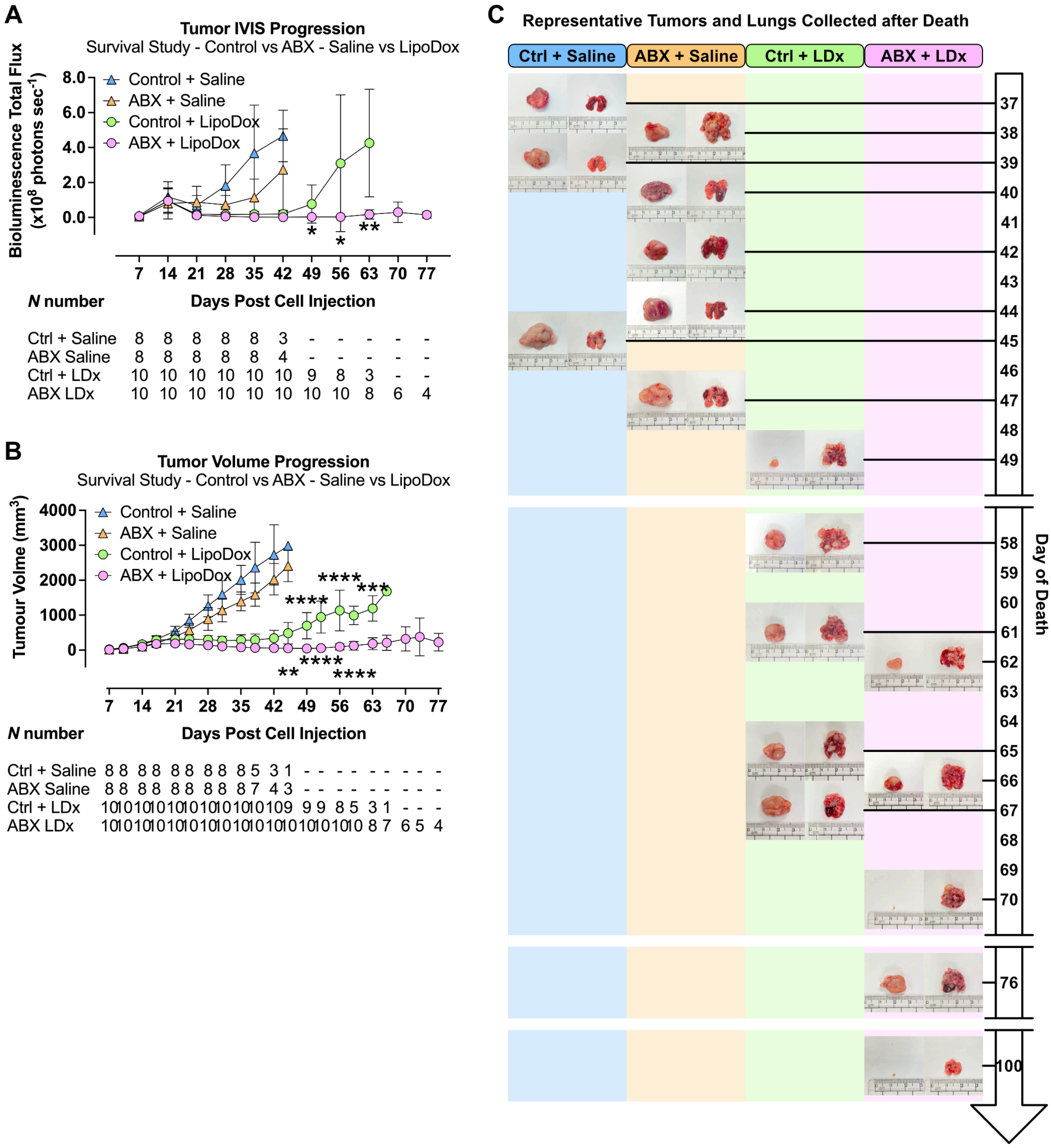

Figure S5. Tumor progression during survival study. (**A**) Progression of tumor growth as measured through quantification of IVIS bioluminescence intensity. Each piece of data from day 7 to day 42 was analyzed individually with one-way analysis of variance with Tukey's correction, while each piece of data from day 49 to day 63 was analyzed individually with two-tailed unpaired *t* test. (**B**) Progression of tumor growth as measured through volume measurement using calipers. Each piece of data from day 7 to day 45 was analyzed individually with one-way analysis of variance with Tukey's correction, while each piece of data from day 49 to day 63 was analyzed individually with two-tailed unpaired *t* test. (**C**) Images of tumor and lungs from different groups taken on the day of

death of each mouse. For both IVIS and tumor volume data, when the number of mice for a group dropped to below three, that particular group was excluded from the statistical analysis. The statistical significance shown on the graphs were between control + LipoDox and ABX + LipoDox groups. $^{*}P < 0.05$; $^{**}P < 0.01$; $^{***}P < 0.001$.

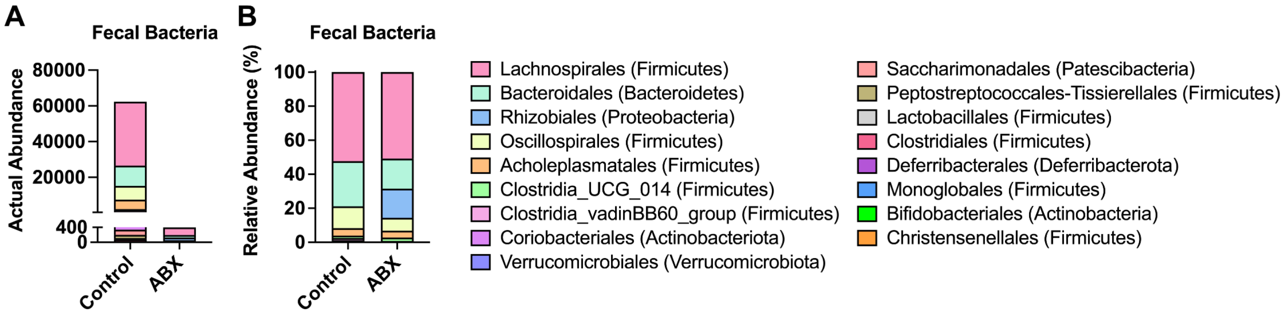

Figure S6. 16S V3-V4 next generation sequencing of fecal samples. (**A**) Absolute abundance of fecal bacteria population. (**B**) Relative abundance of fecal bacteria population.

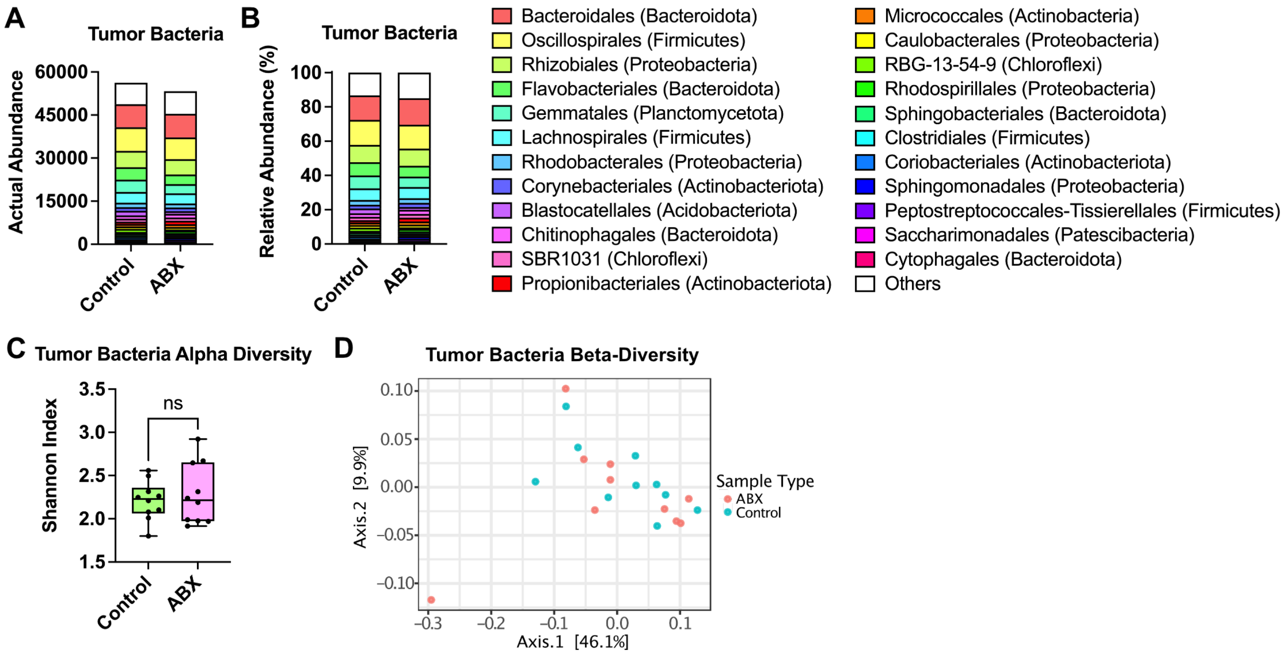

Figure S7. 16S V3-V4 next generation sequencing of tumor samples. (**A**) Absolute abundance of tumor bacteria population. (**B**) Relative abundance of tumor bacteria population. (**C**) Shannon alpha diversity of tumor bacteria. (**D**) Beta diversity of tumor bacteria. Mann-Whitney test was used to analyze the data in (**C**). ns, not significant.

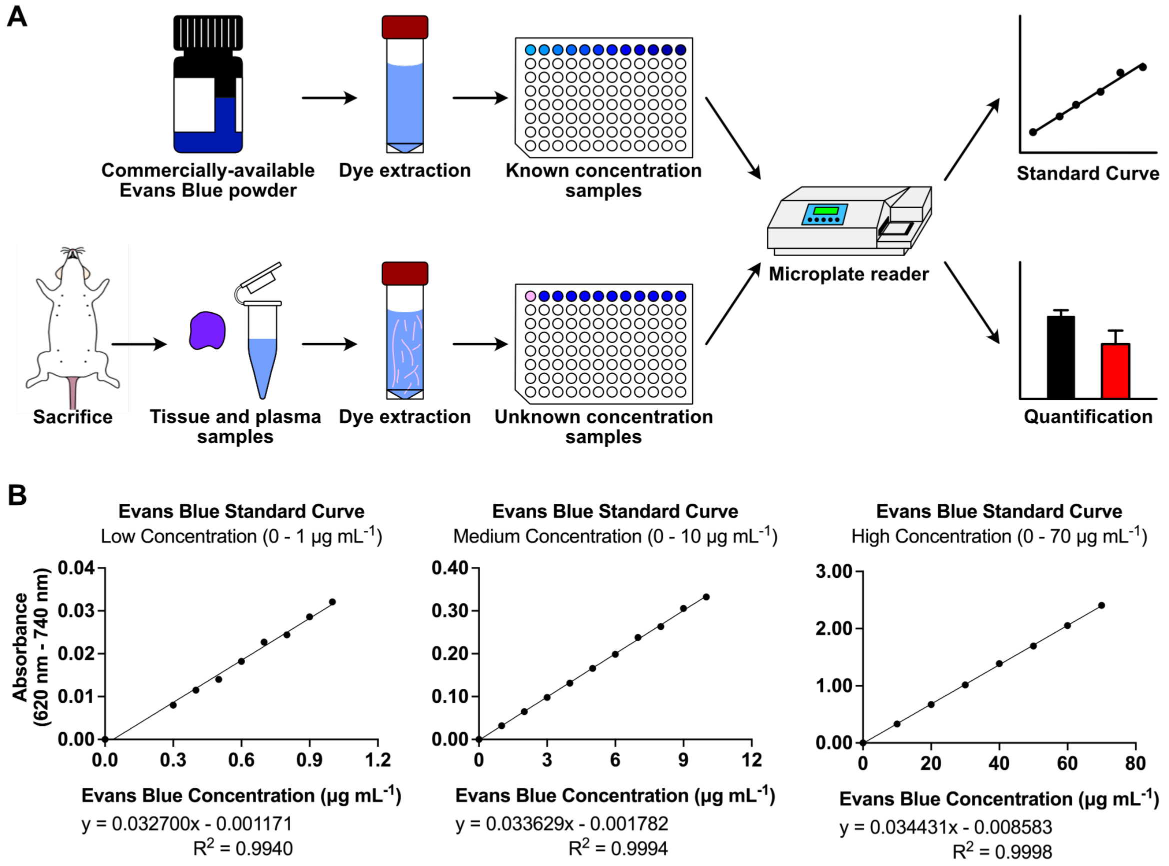

Figure S8. Standard curves of Evans Blue. (**A**) A serial dilution of Evans Blue dye was prepared and their absorbance was measured by a microplate reader to construct standard curves which were then used to quantify the dye content in tissue and plasma samples. (**B**) Standard curves for Evans Blue.

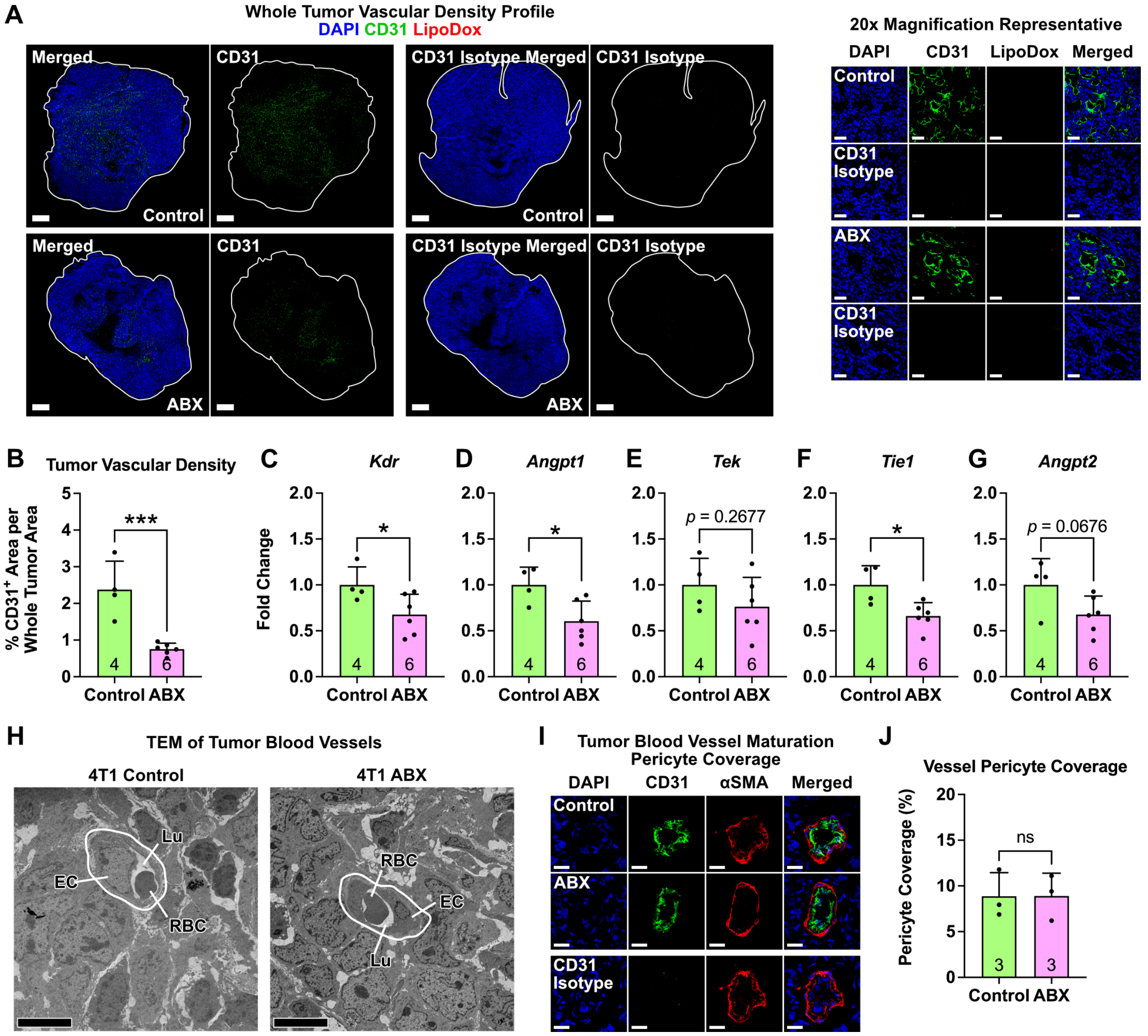

Figure S9. Effects of gut microbiota depletion on tumor vasculature. (**A**) Representative confocal laser scanning microscopy images of whole tumors and 20x magnification stained with endothelial cell marker CD31. Scale bar for whole tumor images: 1 mm; scale bar for 20x magnification images: 50 μm. (**B**) Quantification of tumor vascular density obtained from whole tumor images. (**C-G**) Gene expression of angiogenesis regulators in tumor (**C**) *Kdr*, (**D**) *Angpt1*, (**E**) *Tek*, (**F**) *Tie1* and (**G**) *Angpt2* following gut microbiota depletion. (**H**) TEM images of tumor blood vessels. Scale bar: 8 μm. EC: endothelial cells, R: red blood cells, Lu: lumen. (**I**) Representative confocal laser scanning microscopy images of tumor samples stained for blood vessel (CD31, green) and pericyte (αSMA, red) markers. Scale bar: 20 μm. (**J**) Quantification of pericyte coverage. $^{*}P < 0.05$; $^{***}P < 0.001$; ns, not significant.

Table S1. Diameter and Zeta Potential of Nanoparticles Used in This Study

Polystyrene Nanoparticle Size	Diameter (nm)		Zeta Potential (mV)	
	Non-PEGylated	PEGylated	Non-PEGylated	PEGylated
20 nm	21.0 ± 1.3	29.0 ± 1.2	–40.0 ± 5.5	–5.5 ± 0.8
100 nm	93.8 ± 3.2	105.7 ± 7.4	–30.3 ± 3.7	–3.8 ± 1.6
1000 nm	1099 ± 41	1126 ± 84	–26.0 ± 1.2	–2.0 ± 0.9

Nanoparticle	Diameter (nm)	Zeta Potential (mV)
LipoDox	97.11 ± 0.65	–0.88 ± 0.41

Table S2. Primer Sequences for RT-qPCR Analyses

Stool RT-qPCR	Forward	Reverse
16S rDNA V3-V4	TCCTACGGGAGGCAGCAGT	GGACTACCAGGGTATCTAATCCTGTT

Tumor RT-qPCR		Forward	Reverse
Protein	Gene		
ZO-1	*Tjp1*	CCTGTGAAGCGTCACTGTGT	CGCGGAGAGAGACAAGATGT
Ocln	*Ocln*	CATAGTCAGATGGGGGTGGA	ATTTATGATGAACAGCCCCC
JAM-A	*F11r*	AGTGTACACCGAACCCTTGC	TGTAACTGTAATGGGCACCG
VE-Cadherin	*Cdh5*	TCCTCTGCATCCTCACTATCACA	GTAAGTGACCAACTGCTCGTGAAT
CD31	*Pecam-1*	CCAAAGCCAGTAGCATCATGGTC	GGATGGTGAAGTTGGCTACAGG
VEGFR2	*Kdr*	CGAGACCATTGAAGTGACTTGCC	TTCCTCACCCTGCGGATAGTCA
Angpt-1	*Angpt1*	AACCGAGCCTACTCACAGTACG	GCATCCTTCGTGCTGAAATCGG
Angpt-2	*Angpt2*	AACTCGCTCCTTCAGAAGCAGC	TTCCGCACAGTCTCTGAAGGTG
Tie-1	*Tie1*	CAAGGTCACACACACGGTGAA	GCCAGTCTAGGGTATTGAAGTAGG
Tie-2	*Tek*	ATGTGGAAGTCGAGAGGCGAT	CGAATAGCCATCCACTATTGTCC
GAPDH	*Gapdh*	ACCCAGAAGACTGTGGATGG	CACATTGGGGGTAGGAACAC
